# Fasting hepatic insulin clearance reflects postprandial hepatic insulin clearance: a brief report

**DOI:** 10.1186/s13098-023-01241-4

**Published:** 2023-12-20

**Authors:** Tsuyoshi Okura, Risa Nakamura, Sonoko Kitao, Yuichi Ito, Mari Anno, Kazuhisa Matsumoto, Kyoko Shoji, Kazuhiko Matsuzawa, Shoichiro Izawa, Hiroko Okura, Etsuko Ueta, Masahiko Kato, Takeshi Imamura, Shin-ichi Taniguchi, Kazuhiro Yamamoto

**Affiliations:** 1 Division of Cardiovascular Medicine, Endocrinology and Metabolism, Tottori University Faculty of Medicine, 36-1 Nishi-cho, Yonago, Tottori 683-8504 Japan; 2 School of Health Science, Tottori University Faculty of Medicine, Yonago, Tottori Japan; 3 Division of Molecular Pharmacology, Tottori University Faculty of Medicine, Yonago, Tottori Japan; 4 Department of Community-based Family Medicine, Tottori University Faculty of Medicine, Yonago, Tottori Japan

**Keywords:** Insulin, Clearance and action, Type 2 diabetes, Insulin clamp

## Abstract

**Background:**

Hepatic insulin clearance (HIC) is an important pathophysiology of type 2 diabetes mellitus (T2DM). HIC was reported to decrease in patients with type 2 diabetes and metabolic syndrome. HIC is originally calculated by post-load insulin and C-peptide from the oral glucose tolerance test (OGTT). However, OGTT or meal tolerance tests are a burden for patients, and OGTT is not suitable for overt diabetes due to the risk of hyperglycemia. If we can calculate the HIC from the fasting state, it is preferable. We hypothesized that fasting HIC correlates with postprandial HIC in both participants with T2DM and without diabetes. We investigated whether fasting HIC correlates with postprandial HIC in overt T2DM and nondiabetes subjects (non-DM) evaluated by using glucose clamp and meal load.

**Methods:**

We performed a meal tolerance test and hyperinsulinemic–euglycemic clamp in 70 subjects, 31 patients with T2DM and 39 non-DM subjects. We calculated the postprandial C-peptide AUC-to-insulin AUC ratio as the postprandial HIC and the fasting C-peptide-to-insulin ratio as the fasting HIC. We also calculated whole-body insulin clearance from the glucose clamp test.

**Results:**

The fasting HIC significantly correlated with postprandial HIC in T2DM (*r_S* = 0.82, P < 0.001). Nondiabetes subjects also showed a significant correlation between fasting and postprandial HIC (*r_S* = 0.71, P < 0.001). Fasting HIC in T2DM was correlated with BMI, HbA1c, gamma-glutamyl transpeptidase, HOMA-IR, HOMA-beta, M/I, and whole-body insulin clearance. Fasting HIC in nondiabetes subjects was correlated with HOMA-IR and HOMA-beta.

**Conclusions:**

These results suggest that fasting HIC is strongly correlated with postprandial HIC in both overt T2DM and non-DM patients, as evaluated by the meal test and glucose clamp method. Fasting HIC could be a convenient marker of HIC.

## Background

The pathophysiology of type 2 diabetes mellitus (T2DM) consists of insulin resistance and relatively decreased insulin secretion [1]. Hepatic insulin clearance (HIC) is considered an important pathophysiology of hyperinsulinemia, metabolic syndrome, and type 2 diabetes [2]. Some studies reported that hepatic insulin clearance was decreased in patients with type 2 diabetes [3]. However, hepatic insulin clearance increased with glucose infusion by direct hepatic vein sampling in healthy humans in a previous study [4]. In addition, another clinical and basic study suggested that decreased zinc secretion from β-cells enhances insulin clearance in the liver, leading to the onset of high blood glucose [5]. This study showed that humans with the solute carrier family 30-member 8 (SLC30A8) risk allele and ZnT8 knockout mice have increased hepatic insulin clearance, which was assessed using the C-peptide-to-insulin ratio and postprandial hyperglycemia [5]. We previously reported that hepatic insulin clearance is increased in patients with high HbA1c type 2 diabetes [6]. These results suggest that patients with diabetes and hyperglycemia have dysregulated hepatic insulin clearance.

HIC is originally calculated by post-load insulin and C-peptide from the oral glucose tolerance test (OGTT) [7]. However, it is best to avoid OGTTs in patients with severe diabetes because of the risk of hyperglycemia. Therefore, we used the meal tolerance test (MTT) in a previous study [6]. However, OGTT or meal tolerance tests are a burden for patients. In addition, hepatic insulin clearance increased by 2.5-fold during glucose infusion compared with the fasting state [4]. Furthermore, a previous study reported that the fasting C-peptide/insulin ratio did not correlate with the metabolic clearance rate (MCR) from the euglycemic clamp, but it correlated with the first-phase C-peptide–to–insulin ratio from the hyperglycemic clamp; it may be a useful surrogate of hepatic insulin clearance but not of total insulin clearance [8]. Mathematical models of insulin secretion and kinetics indicate that a 20% inhibition in hepatic clearance is able to remarkably enhance the plasma insulin level following a meal [9]. The postprandial period is inhibited to ensure a sufficient supply of insulin to the peripheral target tissues, and fasting HIC was similar in high HIC and high postprandial glucose model mice ZnT8KO and control mice, but post intraperitoneal glucose tolerance test (IPGTT) HIC was significantly higher in ZnT8KO mice than in control mice [5]. These results suggested that postprandial HIC is more important than fasting HIC in diabetes conditions. If we can calculate the HIC from the fasting state, it is preferable. In addition, few reports have investigated the correlation between fasting HIC and postprandial HIC in both participants with overt type 2 diabetes and those without diabetes in the same study. Insulin clearance consists of hepatic insulin clearance and whole-body insulin clearance, including kidney, skeletal muscle, and adipose tissue [10]. The glucose clamp test is needed to evaluate whole-body insulin clearance, and the OGTT is needed for hepatic insulin clearance. However, the OGTT is not suitable for overt T2DM patients due to the risk of hyperglycemia, and the meal test is preferable for overt T2DM. Therefore, we performed a glucose clamp test and meal tolerance test (MTT) to evaluate whole-body insulin clearance and hepatic insulin clearance in overt type 2 diabetes. We hypothesized that fasting HIC correlates with postprandial HIC in both participants with T2DM and without diabetes. We can evaluate HIC by using fasting HIC without OGTT or MTT, if we can confirm fasting HIC correlates with postprandial HIC in both participants with T2DM and without diabetes. We investigated whether fasting HIC correlates with postprandial HIC in both participants with overt type 2 diabetes and without diabetes evaluated by using glucose clamp and meal load.

## Methods

### Subjects

Thirty-one patients with T2DM and 39 participants without diabetes (non-DM) subjects joined this study at Tottori University Hospital from 2013 to 2022. T2DM was diagnosed using the World Health Organization criteria [11]. We excluded patients from this study with malignancy, pancreatitis, viral hepatitis B and C, liver cirrhosis, renal dysfunction (Cr > 1.3 mg/dl), or those taking corticosteroids or other diabetogenic medications. All participants were on diet therapy alone without medication for diabetes and were of Japanese ethnicity. This study was a cross-sectional study.

We performed this study under the principles of the Declaration of Helsinki. The Ethics Committee of the Faculty of Medicine, Tottori University approved this study (approval number G161). We obtained informed consent from all of the participants using a procedure approved by the Ethics Committee.

### Meal tolerance test (MTT)

The meal tolerance test was performed as we previously reported [6]. Briefly, the participants visited our hospital after overnight fasting and consumed a test meal devised by the Japan Diabetes Society (460 kcal, 50% carbohydrate, 35% fat, 15% protein, and 1.6 g salt). Plasma glucose, serum insulin, and serum C-peptide were measured at 0, 30, 60, and 120 min after the test meal. Plasma glucose was measured using the glucose oxidase method, and serum insulin and C-peptide levels were measured using chemiluminescent immunoassays (human insulin and C-peptide chemiluminescent immunoassay kits; Kyowa Medix, Tokyo, Japan). HbA1c was measured using high-performance liquid chromatography. We converted HbA1c percentage values into International Federation of Clinical Chemistry values (mmol/mol) using the HbA1c converter from the National Institutes of Diabetes and Digestive and Kidney Diseases [12].

Insulin resistance was calculated as follows: Homeostatic model assessment insulin resistance (HOMA-IR) [13] = [fasting plasma glucose (mmol/L)] × [fasting plasma insulin (pmol/L)]/135.

Homeostasis model assessment of beta cell function (HOMA-beta) = {20 × [fasting plasma insulin (pmol/L)]}/{[fasting plasma glucose (mmol/L)] − 3.5} [13].

HOMA2-IR and HOMA2-beta were calculated by HOMA2 calculator [14].

We calculated HOMA2, T2DM 28 participants, and Non-DM 36 participants, because the HOMA-2 calculator could not calculate if insulin levels were under 20 pmol/L.

HIC was calculated as follows:

Postprandial HIC was calculated by the ratio of the incremental areas under the meal tolerance test (MTT) curve (area under the curve [AUC]) C-peptide 0–120 min/AUC insulin 0–120 min) [15]. We calculated the AUC using the trapezoidal method. The postprandial HIC was calculated with all the OGTT timepoints (0, 30, 60 and 120 min).

Fasting HIC was calculated by the ratio of the (fasting C-peptide 0 min)/(fasting insulin 0 min) [15].

#### Measurements of glucose effectiveness

We calculated glucose effectiveness (Sg) by using the difference between glycemia at 60 min after the meal and fasting glycemia (G60-G0), and formula Sg = 2.921e^-0.185(G 60-G 0 )^ [16].

### Hyperinsulinemic–euglycemic clamp

We performed the glucose clamp test as previously reported [6]. Briefly, we performed hyperinsulinemic–euglycemic clamp using an artificial endocrine pancreas (STG 55; Nikkiso, Shizuoka, Japan) to evaluate insulin resistance. The protocol involving a primed constant infusion of insulin (100 mU/m^2^/min) was used, and we maintained the plasma glucose levels at 5.2 mmol/L (95 mg/dL). Based on previous studies, this method achieved a steady-state plasma insulin level of 1200 pmol/L in patients with type 2 diabetes [17]. We measured the steady-state glucose infusion rate (GIR) from 90 to 120 min; we defined the mean GIR during this time as the glucose disposal rate (GDR), which is a marker of peripheral insulin resistance. We also calculated the M/I ratio as a measure of the quantity of glucose metabolized per unit of plasma insulin concentration, and we defined the M value as the GDR and the I value as the steady-state insulin concentration [18]. Whole-body insulin clearance was calculated as the insulin infusion rate divided by the steady-state insulin concentration [18].

### Statistical analysis

We expressed the data as the mean ± standard deviation. We assessed differences in the mean value of clinical parameters between T2DM and non-DM participants using the Mann-Whitney U test. The chi-square test was used for categorical comparisons of sex data. The correlations between parametric clinical variables and HIC were determined using Spearman’s correlation analysis.

We considered values of *P* < 0.05 to be significant. We used PRISM9 software (GraphPad Software, San Diego, USA) for analyses.

A power analysis was performed to compare the correlation between fasting HIC and postprandial HIC using SPSS software V.28.0 (SPSS, Chicago, Illinois, USA).

## Results

**Fig. 1:**
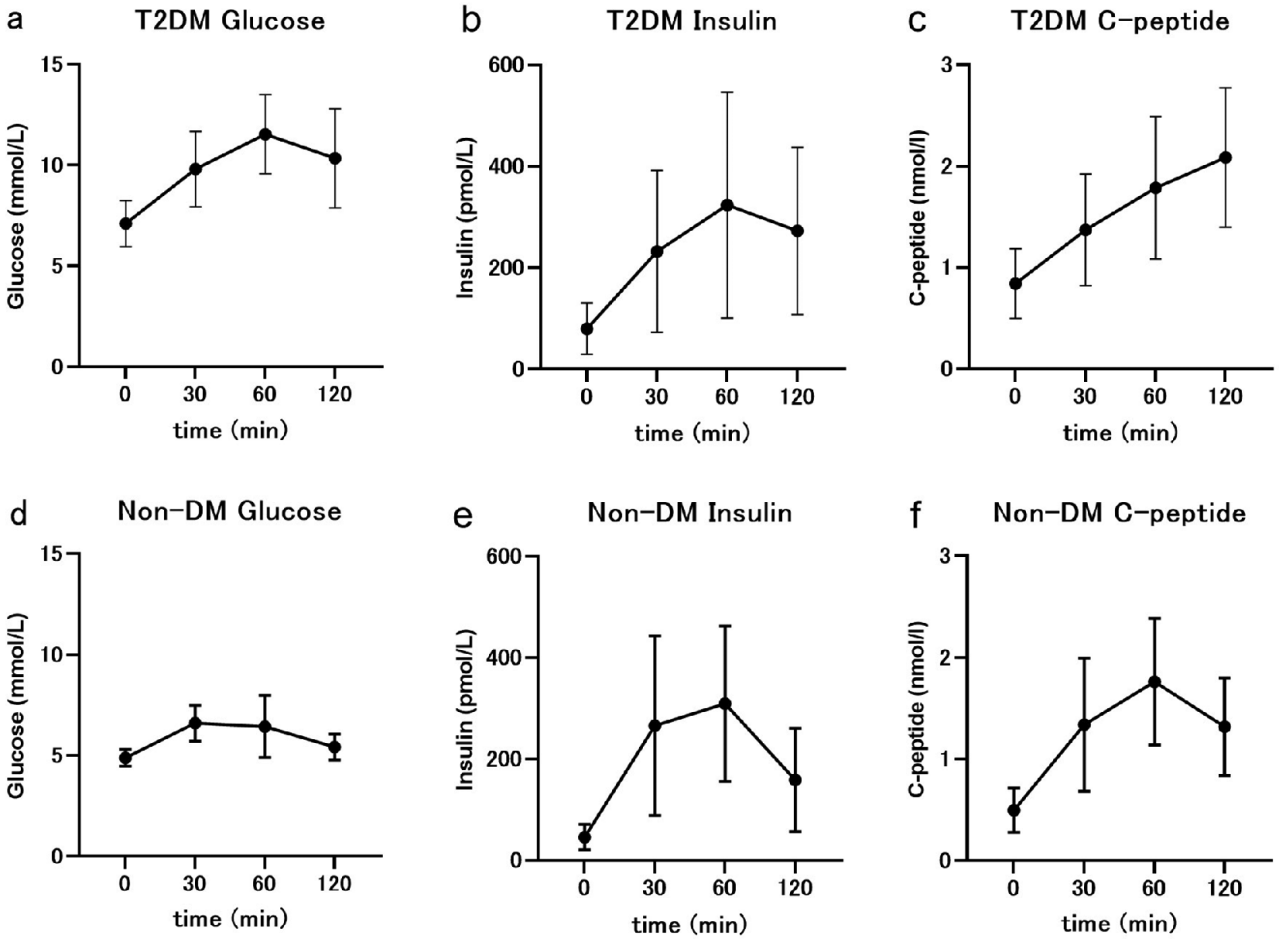
The meal tolerance test (MTT) results. The meal tolerance test (MTT) results. (**a**) Glucose levels after MTT in type 2 diabetes mellitus (T2DM), (**b**) insulin, (**c**) C-peptide, (**d**) glucose levels after MTT in healthy subjects without diabetes mellitus (Non-DM), (**e**) insulin, (**f**) C-peptide

Table 1 shows participant characteristics. Figure 1 shows the MTT results. The T2DM group showed significantly higher age, BMI, glucose levels (fasting, and HbA1c), liver functions (AST, ALT, Gamma-glutamyl transpeptidase (GGTP)), insulin resistance (HOMA-IR, HOMA2-IR) than the non-DM group. The T2DM group also showed significantly lower eGFR, beta cell function (HOMA-beta, HOMA2-beta), glucose effectiveness (Sg), and insulin sensitivity in the glucose clamp (GDR, M/1) than the non-DM group. The postprandial HIC in T2DM tended to show higher than non-DM, whereas there were no significant differences in postprandial HIC, fasting HIC, and whole-body insulin clearance between T2DM and non-DM groups.

**Table 1:** Participant characteristics

Parameters	T2DM	Non-DM	p-value
n	31	39	
Sex (male/female)	16/15	24/15	0.34
Age (years)	57.9 ± 11.3	32.3 ± 8.2	< 0.001
Duration of diabetes (years)	3.8 ± 4.6	-	
BMI (kg/m^2^)	27.1 ± 4.3	21.8 ± 3.0	< 0.001
Waist circumstance (cm)	94.1 ± 11.8	76.8 ± 10.0	< 0.001
Fasting plasma glucose (mmol/L)	7.1 ± 1.1	4.8 ± 0.4	< 0.001
HbA1c (%)	7.7 ± 1.4	5.3 ± 0.3	< 0.001
HbA1c (mmol/mol)	60 ± 15	34 ± 3	< 0.001
AST (IU/L)	33.4 ± 20.3	21.2 ± 8.9	< 0.005
ALT (IU/L)	50.4 ± 35.4	25.7 ± 24.8	< 0.005
Gamma-GTP (IU/L)	54.1 ± 50.2	23.1 ± 12.1	< 0.005
Fasting insulin (pmol/L)	80.0 ± 51.0	45.7 ± 25.2	< 0.001
Fasting C-peptide (nmol/L)	0.80 ± 0.30	0.49 ± 0.21	< 0.001
Postprandial HIC (L·min^–1^·m^2^)	8.2 ± 3.8	6.7 ± 2.1	0.06
Fasting HIC (L·min^–1^·m^2^)	14.2 ± 9.2	12.2 ± 4.7	0.23
eGFR (mL/min/1.73 m^2^)	85.9 ± 20.2	97.1 ± 14.0	< 0.05
Creatinine (mg/dL)	0.70 ± 0.20	0.70 ± 0.15	0.50
HOMA-beta (%)	80.0 ± 54.8	114.3 ± 60.8	< 0.05
HOMA2-beta (%)	66.2 ± 32.8	90.3 ± 28.2	< 0.05
HOMA-IR	4.2 ± 2.7	1.68 ± 1.0	< 0.001
HOMA2-IR	1.62 ± 0.96	0.89 ± 0.46	< 0.001
Sg (min^− 1^ × 10^− 2^)	1.32 ± 0.29	2.28 ± 0.78	< 0.001
GDR (mg/kg/min)	6.0 ± 1.9	10.0 ± 2.8	< 0.001
M/I (100 × mg · kg^− 1^ · body weight^− 1^ · min^− 1^ · mU^− 1^ · l^− 1^)	7.3 ± 6.4	11.6 ± 5.2	< 0.005
Whole body insulin clearance (mL/min)	1.6 ± 0.8	2.3 ± 4.1	0.18

Data are presented as the mean ± standard deviation. (All n = T2DM 31, Non-DM 39; HOMA2 beta and IR, n = T2DM 28, Non-DM 36)

ALT, alanine aminotransferase; AST, aspartate aminotransferase; BMI, body mass index; GDR, glucose disposal rate; GGTP, gamma-glutamyl transpeptidase; HbA1c, glycated hemoglobin; HOMA-beta, homeostatic model assessment beta cell function; HOMA-IR, homeostasis model assessment for insulin resistance; Sg, glucose effectiveness

**Fig. 2:**
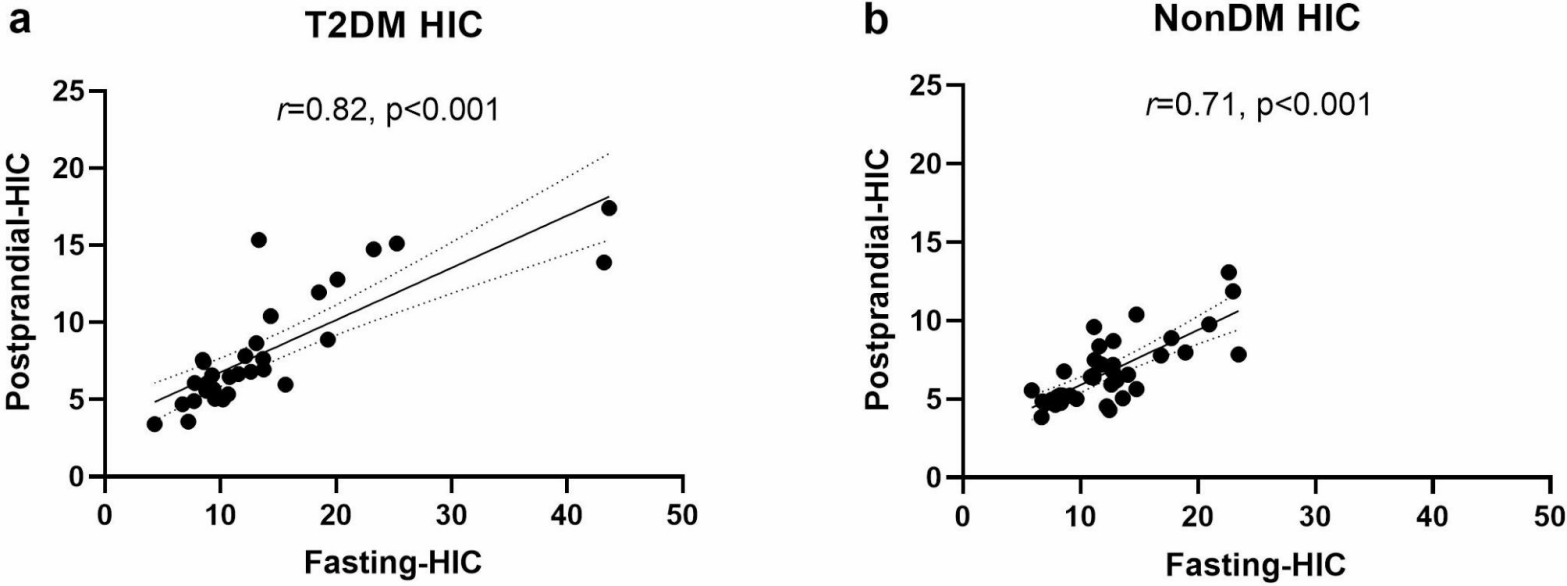
Correlation coefficients between fasting and postprandial hepatic insulin clearance. Correlation coefficients were determined using Spearman’s product-moment correlation coefficient test

The correlation between fasting and postprandial HIC is shown in Fig. 2. The fasting HIC significantly correlated with postprandial HIC in T2DM (*r_S* = 0.82, P < 0.001). Non-DM subjects also showed a significant correlation between fasting HIC and postprandial HIC (*r_S* = 0.71, P < 0.001).

A power analysis was performed to compare the correlation between fasting HIC and postprandial HIC. The *r* values in the correlation between fasting HIC and postprandial HIC were 0.82 in T2DM (n = 31) and 0.71 (n = 39) in non-DM. The estimated power was > 99% in both T2DM and non-DM patients.

The correlations between fasting HIC and clinical parameters are shown in Table 2. Fasting HIC in T2DM was correlated with BMI, HbA1c, GGTP, HOMA-IR, HOMA2-IR, HOMA-beta, HOMA2-beta, GDR, M/I, and whole-body insulin clearance. Fasting HIC in non-DM patients was correlated with HOMA-IR, HOMA2-IR, HOMA-beta, and HOMA2-beta. Fasting HIC did not correlate with glucose effectiveness (Sg) in both T2DM and non-DM.

**Table 2:** Correlation between the fasting HIC and clinical parameters

Parameters	T2DM		Non-DM	
	*r*-value	P value	*r*-value	P value
Age (years)	0.27	0.07	-0.19	N.S.
Duration of diabetes (years)	0.05	N.S.	-	
BMI (kg/m^2^)	-0.58	< 0.0005	-0.15	N.S.
Waist circumstance (cm)	-0.55	< 0.001	-0.10	N.S.
Fasting plasma glucose (mmol/L)	0.14	0.22	0.05	N.S.
HbA1c (%)	0.49	< 0.05	0.25	0.06
AST (IU/L)	-0.27	0.06	0.18	N.S.
ALT (IU/L)	-0.27	0.08	0.02	N.S.
Gamma-GTP (IU/L)	-0.37	< 0.05	0.05	N.S.
Fasting insulin (pmol/L)	-0.82	< 0.0001	-0.67	< 0.0001
Fasting C-peptide (nmol/L)	-0.42	< 0.01	-0.004	N.S.
eGFR (mL/min/1.73 m^2^)	-0.08	N.S.	0.13	N.S.
Creatinine (mg/dL)	0.06	N.S.	-0.15	N.S.
HOMA-beta (%)	-0.84	< 0.0001	-0.41	< 0.05
HOMA2-beta (%)	-0.77	< 0.0001	-0.64	< 0.0001
HOMA-IR	-0.77	< 0.0001	-0.62	< 0.0001
HOMA2-IR	-0.80	< 0.0001	-0.57	< 0.0001
Sg (min^− 1^ × 10^− 2^)	0.13	N.S.	0.01	N.S
GDR (mg/kg/min)	0.38	< 0.05	0.09	N.S.
M/I ((100 × mg · kg^− 1^ · body weight^− 1^ · min^− 1^ · mU^− 1^ · l^− 1^))	0.56	< 0.0005	0.22	N.S.
Whole-body insulin clearance (mL/min)	0.35	< 0.05	-0.01	N.S.

Correlation coefficients were determined using Spearman’s product moment correlation coefficient test. (all n = T2DM 31, Non-DM 39; HOMA2 beta and IR, n = T2DM 28, Non-DM 36)

ALT, alanine aminotransferase; AST, aspartate aminotransferase; BMI, body mass index; GDR, glucose disposal rate; GGTP, gamma-glutamyl transpeptidase; HbA1c, glycated hemoglobin; HOMA-beta, homeostatic model assessment beta cell function; HOMA-IR, homeostasis model assessment for insulin resistance; N.S., not significant; Sg, glucose effectiveness

Table 3 shows the comparison between BMI under 25 and over 25 in Type 2 DM. T2DM patients with a BMI under 25 showed significantly higher fasting HIC, postprandial HIC, and HbA1c than T2DM patients with a BMI over 25. T2DM patients with a BMI under 25 also showed significantly lower insulin secretion (HOMA-beta, HOMA2-beta) and lower insulin resistance (HOMA-IR, HOMA2-IR, M/I) than T2DM patients with a BMI over 25. The fasting glucose and fasting C-peptide, glucose effectiveness (Sg), and whole-body insulin clearance levels were the same between BMIs under 25 and over 25 in T2DM.

**Table 3:** Participant characteristics Type 2 DM BMI under 25 and over 25

Parameters	BMI < 25	BMI > 25	P value
n	9	22	
Sex (male/female)	4/5	11/11	
Age (years)	58.6 ± 9.7	57.6 ± 12.1	0.82
Duration of diabetes (years)	4.0 ± 5.1	3.2 ± 4.0	0.69
BMI (kg/m^2^)	22.0 ± 1.6	29.2 ± 3.2	< 0.001
Waist circumstance (cm)	81.3 ± 7.9	99.5 ± 8.6	< 0.001
Fasting plasma glucose (mmol/L)	7.60 ± 1.80	6.90 ± 0.70	0.23
HbA1c (%)	8.9 ± 1.4	7.3 ± 0.8	< 0.05
HbA1c (mmol/mol)	73 ± 16	56 ± 9	
AST (IU/L)	31.0 ± 25.5	34.4 ± 18.4	0.73
ALT (IU/L)	39.1 ± 29.8	55.0 ± 37.1	0.22
Gamma-GTP (IU/L)	44.9 ± 55.3	57.9 ± 48.8	0.55
Fasting insulin (pmol/L)	38.8 ± 32.0	96.4 ± 47.9	< 0.001
Fasting C-peptide (nmol/L)	0.70 ± 0.30	0.90 ± 0.30	0.09
Postprandial HIC (L·min^–1^·m^2^)	12.8 ± 3.4	6.3 ± 1.9	< 0.001
Fasting HIC (L·min^–1^·m^2^)	23.2 ± 12.4	10.6 ± 3.7	< 0.05
eGFR (mL/min/1.73 m2)	90.4 ± 14.8	84.0 ± 22.0	0.36
Creatinine (mg/dL)	0.60 ± 0.10	0.70 ± 0.20	0.34
HOMA-beta (%)	31.1 ± 18.7	99.9 ± 52.2	< 0.001
HOMA2-beta (%)	35.4 ± 13.3	76.4 ± 30.8	< 0.001
HOMA-IR	2.5 ± 2.4	5.0 ± 2.6	< 0.05
HOMA2-IR	1.0 ± 0.7	1.8 ± 0.9	< 0.05
Sg (min^− 1^ × 10^− 2^)	1.25 ± 0.33	1.34 ± 0.26	0.44
GDR (mg/kg/min)	6.9 ± 1.7	5.7 ± 1.9	0.09
M/I (100 × mg · kg^− 1^ · body weight^− 1^ · min^− 1^ · mU^− 1^ · l^− 1^)	12.9 ± 9.2	5.1 ± 2.8	< 0.05
Whole-body insulin clearance (mL/min)	2.1 ± 1.4	1.4 ± 0.3	0.13

Data are presented as the mean ± standard deviation. (HOMA2 beta and IR, n = BMI < 25, 7; BMI > 25, 22)

ALT, alanine aminotransferase; AST, aspartate aminotransferase; BMI, body mass index; GDR, glucose disposal rate; GGTP, gamma-glutamyl transpeptidase; HbA1c, glycated hemoglobin; HOMA-beta, homeostatic model assessment beta cell function; HOMA-IR, homeostasis model assessment for insulin resistance; Sg, glucose effectiveness

## Discussion

This study showed that fasting HIC is strongly correlated with postprandial HIC in both participants with type 2 diabetes and without diabetes, and fasting HIC could be a convenient marker of HIC. We consider it important that fasting HIC strongly correlated with postprandial HIC in both participants with overt type 2 diabetes and those without diabetes in the same study. In addition, T2DM patients with a BMI under 25 showed significantly higher fasting and postprandial HIC than T2DM patients with a BMI over 25. T2DM patients with a BMI under 25 also showed significantly lower insulin secretion and lower insulin resistance than T2DM patients with a BMI over 25. However, fasting glucose and fasting C-peptide, and whole-body insulin clearance were at the same levels between BMIs under 25 and over 25 in T2DM. These results suggested that lean T2DM patients have higher hepatic insulin clearance than obese T2DM patients. These results are important for the pathophysiology of T2DM.

T2DM showed lower glucose effectiveness than non-DM, but glucose effectiveness did not correlate with fasting HIC, and did not show significant differences between BMI < 25 and > 25 in T2DM. The postprandial HIC in T2DM tended to show higher than non-DM, whereas there were no significant differences in postprandial HIC, fasting HIC, and whole-body insulin clearance between T2DM and non-DM groups. The fasting HIC correlated with BMI, HbA1c, and whole-body insulin clearance in the T2DM. These results suggested that fasting HIC is affected by BMI and HbA1c rather than glucose effectiveness in the diabetes condition.

The fasting HIC was also significantly correlated with HbA1c in T2DM. We previously reported that postprandial HIC was increased in high HbA1c patients with T2DM [6], and T2DM participants showed higher HIC than participants without diabetes [19]. In addition, another hyperglycemic and hyperinsulinemic-euglycemic clamp Japanese study showed that hepatic and peripheral insulin clearance significantly increase and decrease, respectively, from healthy to borderline type and T2DM [20]. Another European 3 years mixed meal study showed that faster HbA1c progression was independently associated with faster deterioration of insulin sensitivity and b-cell glucose sensitivity and increasing insulin clearance [21]. In addition, another OGTT and hyperinsulinemic euglycemic glucose clamp study showed that postprandial insulin clearance (the rate of removal of insulin from plasma) is a function of the amount of insulin delivered to organs that clear insulin, so people with obesity and insulin resistance have low postprandial insulin clearance because of high insulin secretion rate (ISR) and postprandial plasma insulin concentrations, whereas people with obesity and T2DM have “normal” insulin clearance because of defective ISR and lower postprandial insulin concentrations [22]. These results suggested that the cause of low or high insulin clearance is the amount of insulin secretion, i.e., insulin clearance in T2DM being “normal” (high) is attributed to low insulin secretion. In addition, a recent report demonstrated that high blood glucose decreases insulin clearance [23]. Our results might make the increased HIC in type 2 diabetes merely an assessment of decreased insulin secretion. The reasons underlying these results remain elusive; furthermore, studies are needed.

In this study, fasting HIC was significantly correlated with gamma-GTP. We also previously reported that postprandial HIC correlated with GGTP [6], and a similar result was reported by another previous study, where increased gamma-GTP and ALT were observed in healthy individuals with high hepatic insulin resistance and decreased HIC [24].

Moreover, fasting HIC was strongly correlated with HOMA-IR, and fasting HIC was associated with the M/I value. HOMA-IR is mainly associated with hepatic insulin resistance, and glucose clamp is mainly associated with muscle insulin resistance [25]. According to these results, hepatic insulin sensitivity might be associated more with HIC than with muscle insulin sensitivity. However, fasting HIC correlated with whole-body insulin clearance, which might mean that fasting HIC reflects hepatic and peripheral insulin clearance.

We consider our study to have several limitations. Because of the small number of participants, our study requires a larger number of participants. Thus, we decided to add “A Brief Report” to the title. However, the glucose clamp method is a very complicated technique, and it is difficult to enroll patients with T2DM without medication. We are currently continuing this study, and we would like to publish larger results in the future. The controls are much younger, the other issue is that controls are also leaner. Age- and BMI-matched studies are needed. In addition, our study subjects were all Japanese. A previous study reported that African Americans show lower hepatic clearance than non-Hispanic whites [15]. We must consider ethnic differences. We used MTT in our study because we should avoid the risk of hyperglycemia by using OGTTs in patients with severe diabetes. We calculated hepatic clearance from MTT results, and consuming a test meal was different from administering a pure glucose load, which might affect glucose and insulin levels. However, some studies used the HIC that was obtained from MTT, and we consider that our study was acceptable [26]. We also excluded patients with serum creatinine > 1.3 mg/dL based on an earlier report because C-peptide levels are strongly affected by renal function [27]. We must take care when we calculate the HIC using C-peptide in patients with renal dysfunction. We also did not evaluate liver-specific insulin resistance because of the difficulty of using tritium-glucose in Japan. Our glucose clamp protocol maintains the plasma glucose levels at 5.2 mmol/L (95 mg/dL) even the patients with diabetes. This protocol might be low-level for patients with diabetes. As a result, there is a possibility that GDR and M/I values did not reflect accurately the insulin resistance of the patients with diabetes. Despite these limitations, we consider that our study would contribute to our daily clinical work or clinical studies. We previously reported that SGLT2 inhibitor increased postprandial HIC, and decreased hyperinsulinemia, insulin resistance, and liver functions [28]. We consider that our findings contribute to the treatment strategy, because we can estimate postprandial HIC from only one fasting blood sample based on our results.

In conclusion, fasting HIC is strongly correlated with postprandial HIC in both overt T2DM and non-DM by using meal load and glucose clamp, and fasting HIC could be a convenient marker of HIC.

## Data Availability

The datasets supporting the conclusions of this article are included within the article.

## Abbreviations

AUC: area under the curve
eGFR: estimated glomerular filtration rate
GDR: glucose disposal rate
GGTP: gamma-glutamyl transpeptidase
HbA1c: glycated hemoglobin
HIC: hepatic insulin clearance
HOMA-beta: homeostatic model assessment beta cell function
HOMA-IR: homeostasis model assessment for insulin resistance
Sg: glucose effectiveness
